# Micro RNA in Colorectal Cancer—Potential Diagnostic and Prognostic Markers—An Updated Review

**DOI:** 10.3390/ijms26178615

**Published:** 2025-09-04

**Authors:** Weronika Pająk, Jakub Kleinrok, Joanna Pec, Karolina Michno, Jan Wojtas, Miłosz Badach, Barbara Teresińska, Jacek Baj

**Affiliations:** 1 Department of Forensic Medicine, Medical University of Lublin, Jaczewskiego 8b, 20-090 Lublin, Poland; wapajak@gmail.com (W.P.); klejs.90@gmail.com (J.K.); mimbad@interia.pl (M.B.); 2 Department of Correct, Clinical and Imaging Anatomy, Medical University of Lublin, Jaczewskiego 4, 20-090 Lublin, Poland; apec1410@gmail.com (J.P.); jan.wojtas7@gmail.com (J.W.); b.teresinska2@gmail.com (B.T.); 3 Faculty of Medicine, Medical University of Lodz, al. Tadeusza Kosciuszki 4, 90-419 Lodz, Poland; karolina.michno@stud.umed.lodz.pl

**Keywords:** colorectal cancer, diagnostic markers, prognostic markers, microRNA

## Abstract

Colorectal cancer (CRC) is one of the deadliest and most frequently occurring cancers worldwide. Often diagnosed in advanced stages, it requires more challenging treatment. However, emerging studies highlight the possible role of microRNAs (miRNAs) in the screening, diagnosis, and prognosis of CRC. MiRNAs modulate gene expression and can play both roles in tumor suppressors and oncogenes. In CRC, they influence epithelial–mesenchymal transition (EMT), cell proliferation and migration, apoptosis, autophagy, and patients’ treatment response. In clinical applications, they can be used as predictive and prognostic biomarkers as well as for matching the most suitable treatments. Despite its growing popularity, there is still much to discover about their potential usage in medicine.

## 1. Introduction

CRC places itself in the top three most common and malignant cancers worldwide. Posing a significant challenge, it demands a comprehensive treatment approach [1,2,3,4]. Therefore, early detection is crucial, leaving space for researchers to find the most suitable screening, diagnostic, and prognostic markers. During the last decade, multiple studies regarding the possible role of miRNAs have emerged [5]. MiRNAs are short, non-coding sequences of approximately 22 nucleotides, which modulate gene expression. They are present in different biological samples, which makes them more accessible [6]. A great number of studies proved their role in the occurrence and progression of various cancers including CRC, brain cancer, lung cancer, and breast cancer [7,8,9,10,11]. MiRNAs bind to the 3′ untranslated region (3′UTR) of their target genes, therefore they can regulate various functions in cancer cells, acting either as tumor suppressors or oncogenes. They are involved in key cellular processes such as cell proliferation and migration, autophagy, apoptosis, metabolic reprogramming, EMT, and the response to radiation therapy. MiRNAs have already shown potential in clinical oncology as valuable biomarkers. Abnormal expression patterns of miRNAs have been linked to CRC [12]. Moreover, they have been shown to be clinically applicable as early diagnostic biomarkers. The epigenetic regulation of microRNAs, particularly through DNA methylation, has emerged as a significant mechanism in CRC development and progression. Many miRNAs act as tumor suppressors, and their silencing—often via promoter hypermethylation—has been frequently observed in CRC. This reversible nature of DNA methylation highlights miRNAs as promising biomarkers for early detection and prognosis. Aberrant methylation of specific miRNAs, such as miR-124a, miR-137, and miR-34, has been consistently associated with CRC-specific changes, often detectable even in early lesions or non-invasive samples like stool and bowel lavage fluid. These miRNAs not only aid in identifying patients at high risk for CRC, including those with predisposing conditions like ulcerative colitis (UC), but also correlate with tumor differentiation, metastasis, recurrence, and overall survival. Therefore, methylated miRNAs represent a novel and valuable class of epigenetic biomarkers with strong potential for clinical application in CRC diagnostics and patient stratification [13]. Furthermore, in CRC, increasing evidence suggests that dysregulation of specific miRNAs is closely linked to resistance or sensitivity to commonly used chemotherapeutic agents. As such, miRNAs have emerged as promising candidates for use as diagnostic and prognostic biomarkers, as well as potential therapeutic targets aimed at overcoming drug resistance and improving patient outcomes [14]. As an increasing number of studies regarding miRNA emerge, new potential clinical applications are discovered. The aim of this article is to highlight the countless possibilities of clinical miRNAs usage in screening, diagnosis, and predicting CRC, providing updated and comprehensive knowledge. We believe miRNAs to be a vital factor leading to the decrease in CRC mortality and expanding the survival rate of CRC patients. In our work, we have distinguished the most applicable and clinically approved miRNAs.

## 2. Characteristics of MicroRNAs

MiRNAs are an interesting area of research due to their large presence in tissues and body fluids, their ability to influence gene expression, and their potential usefulness as disease markers. Although miRNAs are non-coding RNAs themselves, they can significantly influence informational RNA (mRNA). They specifically affect gene expression levels; therefore, any alterations in their expression can cause changes in the target regulation by affecting cell homeostasis. Such dysregulation has implications for carcinogenesis, among other medical aspects [15].

### 2.1. Biogenesis of MiRNAs

In the process of miRNA biogenesis, its molecules are transcribed as independent units. The formation of miRNAs involves several genes [16]. These include *DROSHA*, *DGCR8* (DiGeorge syndrome critical region 8), *DICER1*, and *AGO1/2*, which encode the Argonaute 1/2 protein. MiRNA processing can occur in two ways: canonical or non-canonical. Most miRNAs undergo canonical biogenesis, which uses enzymes such as the RNase III enzymes, DROSHA, and DICER 1 [17]. The canonical process begins with the generation of a primary microRNA transcript (pri-miRNA), which involves RNA polymerases II or III [16]. Transcription of pri-miRNAs occurs from DNA. The resulting pri-miRNA is then cleaved into a miRNA precursor consisting of approximately 80 nucleotides [18]. This process occurs through the action of RNase III Drosha. Two complexes can be formed to facilitate the separation of the pri-miRNA by RNase III Drosha. The first consists of Drosha and the DGCR8 protein, and the second contains the RNA helicases p72 and p68. Subsequently, the pre-miRNA is transported from the nucleus to the cytoplasm in a GTP-dependent process involving exportin-5, which binds to Ran-GTP-dependent RNA. Furthermore, exportin protects the pre-miRNA from degradation. The transported pre-miRNA is then digested by RNase III and Dicer. This results in the formation of a 22-nt mature miRNA duplex, which develops into two independent strands: a guide strand and a passenger strand. This process depends on ATP. The duplex then unwinds in an ATP-independent process. The passenger strand is usually degraded while the guide strand is loaded into an RNA-induced silencing complex (RISC). The guide strand is conserved and becomes the mature form of the microRNA. A miRISC is formed. This complex includes Dicer, PACT, TRBP, Ago2, and GW182 proteins. The miRISC complex can identify mRNA targets through base complementarity, which gives it the ability to inhibit gene expression [16]. However, some miRNAs are processed in a non-canonical manner. Some are encoded in introns and bypass DROSHA cleavage to form pre-miRNAs directly from excised introns. These pre-miRNAs are then transported to the cytosol, where they are processed into mature miRNAs by DICER1. Non-canonical biogenesis can also occur independently of DICER1. For example, this process occurs during erythropoiesis, when DICER1 levels are naturally low. In this case, the pre-miRNA is processed directly into the mature miRNA by AGO2. The entire process of miRNA biogenesis is strictly controlled at every stage. This is crucial because abnormalities can lead to various diseases, including cancer [17].

### 2.2. Mechanisms of Regulation of Gene Expression by MiRNAs

MiRNA plays pivotal role in many processes through its regulatory functions, such as post-transcriptional regulation of the cell cytoplasm [18]. They can silence mRNA present in the cytoplasm through translation repression, inducing endonuclease cleavage, or by enhancing the process of mRNA deadenylation and decapping [19]. MiRNA can target up to 30% of the human genome, significantly impacting transcriptomes and proteomes [18]. The miRISC in eukaryotes is located in a structure called the processing body, which plays an important role in post-transcriptional silencing involving miRNAs [20]. In miRISC, miRNA acts as a target recognition element, and the Ago protein is a key component. The mammalian genome contains four types of Ago, with Ago2being the most abundant [21]. Ago2 plays a role in mRNA cleavage due to its catalytic function. The other Ago proteins primarily participate in translational repression [22]. Human miRNAs can bind to all Ago proteins, though some show a preference for specific types [21]. MiRNAs target specific sites on messenger mRNAs. These sites are located in the 3′-untranslated region (3′-UTR) [23]. The miRNA exerts its effects in the form of post-transcriptional repression by either degrading the mRNA or inhibiting translation. For mRNA degradation to occur, the action of a 182-kDa protein containing glycine and tryptophan residues (GW182) and the DCP1-DCP2 decapping enzyme complex is required [21,23].

There are three members of the GW182 protein present in human cells. Through these members, GW182 interacts with proteins from the Ago family. GW182 recruits and binds to the cytoplasmic poly(A) tail-binding protein (PABPC), which inhibits the translation of proteins derived from the target mRNA [24]. Subsequently, the polyadenylation tail of mRNA is removed by interacting with the PAN2-PAN3 and CCR4-NOT complexes. The DCP1-DCP2 complex then removes the 5′ cap after shortening the poly(A) tail. This causes rapid degradation of mRNA by the exonuclease XRN1, which acts from the 5′ end [25].

It is assumed that miRNAs can directly participate in gene silencing in the cytoplasm [26]. Notably, miRNAs localized in the cytoplasm can occur in different cellular compartments, such as the endoplasmic reticulum or mitochondria. This is associated with the diversity of their functions and pathways of action [27]. What is more, increasing evidence suggests that they may also be active in the nucleus of the cell. Several miRNAs associated with transcription regulation have been localized in the nucleus [28]. In this location, they may have two functions: one related to transcriptional gene activation (TGA) and the other to transcriptional gene silencing (TGS) [21]. Additionally, miRNAs can regulate gene expression in the cell nucleus, both post-transcriptionally and during transcription. This occurs through interaction with promoter sequences in the form of RNA-RNA, RNA-DNA hybrids, or RNA-DNA triple-helical structures [29]. Furthermore, certain nuclear miRNAs can activate enhancers and initiate gene expression via epigenetic pathways. All of these mechanisms depend on the presence and activity of Ago family proteins [30].

There are complex interrelationships among miRNAs: one can affect many different genes, and the same gene can be regulated by several miRNAs. However, the effect of a single miRNA on a gene is usually moderate. Furthermore, in most cases, the number of mRNA target sites exceeds the number of available miRNA molecules [31]. The ability of miRNAs to recognize targets is closely linked to the presence of an extended seed region consisting of nucleotides 2–8 of the miRNA. Due to seed regions, miRNAs are divided into families [32]. This division is possible based on the presence of identical seed sequences in mature miRNAs. The presence of these sequences allows us to conclude that the given miRNAs will have similar target RNAs [33].

### 2.3. The Role of MiRNAs in Carcinogenesis

MiRNAs appear to play a pivotal role in carcinogenesis, and their contribution to cancer development has been extensively studied. Scientists focus on its involvement in the initiation, growth, and progression of tumors. This is because miRNA can regulate the immune system as well as angiogenesis, cell proliferation, and DNA repair. It can also influence the development of inflammation [34]. Although several miRNAs affecting tumorigenesis have been discovered, their clinical application remains limited due to the difficulty of delivering them efficiently to tumor tissue and their multidirectional effects, which can result in adverse effects beyond the intended target [35].

It is now known that perturbations in miRNA expression play a key role in the formation of cancer, and miRNAs act as negative regulators of their target mRNAs [35]. Many miRNAs are present in regions of the human genome that undergo deletion or amplification associated with tumorigenesis. The data indicates that up to half of the miRNAs may be found in cancer-related areas or in fragile parts of the genome. Although deletions or changes in transcription often affect only a few miRNAs, changes in their transcription can cause significant alterations, including tumorigenesis [36]. Two main groups of miRNAs tightly regulate the function of oncogenes and tumor-suppressor genes in cancer cells: tumor-suppressor miRNAs (TS-miRs), which inhibit tumor growth, and oncomiRs, which promote tumor formation [37]. OncomiRs are overexpressed miRNAs that target genes that inhibit carcinogenesis. They stimulate cell proliferation, angiogenesis, and metastasis [35]. One of these is miR-21, which is overexpressed in many malignancies including lung, bladder, ovarian, and colorectal cancer. In lung cancer, for example, miR-21 influences the PTEN/Akt/GSK3β signaling pathway, promoting tumor growth. What is more, reducing its expression can inhibit tumor development. For example, this occurs in colon cancer through the influence of long non-coding RNAs (lncRNAs) *DGCR5* or *LINC00312* on miR-21 [38].

A group of TS-miRs are miRNAs with reduced expression in tumor cells. Reduced expression of these microRNAs may promote tumor development and progression [39]. Genetic mutations and epigenetic changes lead to the attenuation or loss of TS-miRNA activity and the overstimulation of oncomiRNAs. This results in the overexpression of oncogenes that would normally be inhibited by TS-miRs and the suppression of tumor-suppressor genes by excess oncomiRs [40].

### 2.4. Ways to Detect and Analyze MiRNAs

The important role that miRNAs play in regulating genes and biological functions makes the specific and sensitive detection of miRNAs increasingly important both for basic biological sciences and in connection with the diagnosis and treatment of diseases, including cancer [36]. However, due to the specific characteristics of miRNAs, detecting them poses a challenge. It is worth noting that they are short and highly similar, especially if they belong to the same family. For this reason, the tool used to detect them quantitatively must be highly specific. Furthermore, miRNAs account for only 0.01% of total RNA, and their concentration in blood plasma is in the picomolar range. Therefore, the detection method must be highly sensitive [40].

There are many reference methods for detecting miRNA. Each of them has its own advantages and disadvantages. Traditional methods are used to detect miRNAs, with Northern blotting considered the gold standard. Unfortunately, Northern blotting is time-consuming, requires a large RNA sample, and shows low sensitivity [35]. Microarray-based techniques are distinguished by their high throughput and ability to analyze multiple miRNAs simultaneously. However, this method has limitations as well, including low sensitivity and a long hybridization time. The most commonly used methods for miRNA analysis are quantitative PCR (qPCR) techniques, including quantitative reverse transcription PCR (qRT-PCR) using a stem-loop structure and poly(A)-tailed qRT-PCR. These methods are distinguished by their high specificity and sensitivity. One limitation of these methods is their complexity and the need for specialized laboratory skills. Additionally, there is a risk of false positives during the amplification process [41]. Moreover, newer and newer methods are being used to detect miRNAs. Among them, lateral flow assays (LFAs) are increasingly emerging as rapid tools for detecting miRNAs. Compared to traditional miRNA detection techniques, these tests are simpler, less expensive, and more portable. However, more research is needed to develop LFAs that are sufficiently sensitive and accurate [42].

## 3. MicroRNAs in Inflammatory Colorectal Cancer Genesis

**Figure 1:**
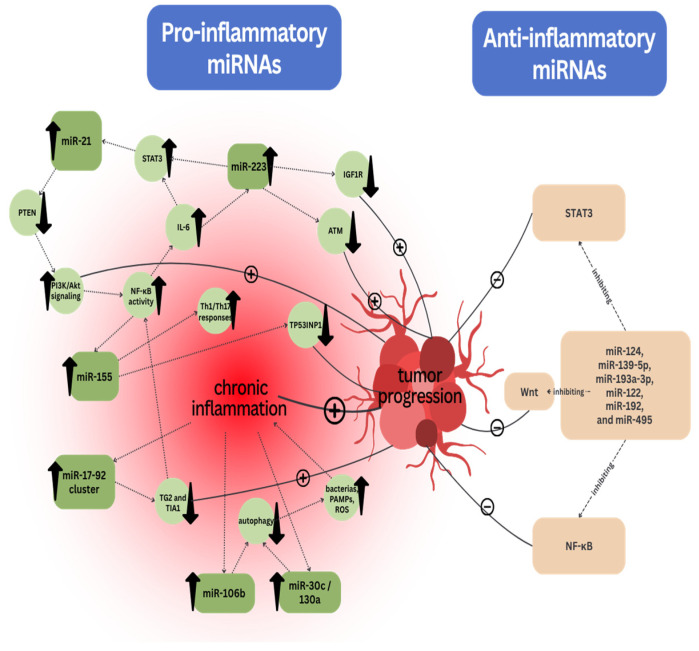
miRNAs in tumor progression.

Chronic inflammation drives tumorigenesis by triggering both tumor initiation and promotion through mechanisms like DNA damage and epigenetic alterations, often mediated by reactive oxygen species (ROS) released by immune cells. Inflammation can also disrupt the intestinal epithelial barrier, allowing exposure to mutagens or pathogenic microorganisms that further promote tumor formation. Pro-inflammatory cytokines, such as IL-6, TNFα, IL-1β, and IFNγ, enhance the activation of key signaling pathways like NF-κB and STAT3, which drive tumor progression. In addition, inflammation induces changes in the tumor microenvironment (TME), fueling immune cell recruitment and contributing to a pro-tumorigenic atmosphere. Tumor-promoting inflammation also leads to immune evasion, where tumor-derived factors suppress the activity of cytotoxic immune cells. Furthermore, epigenetic modifications, such as altered DNA methylation and microRNA regulation, play a critical role in silencing tumor-suppressor genes and driving CRC progression. Tumor-elicited inflammation further complicates the cancer process, creating a feedback loop that sustains tumor growth and metastasis [43,44]. In many studies, it has been stated that miRNAs play a pivotal role in various inflammation signaling pathways. Acting as both pro-inflammatory and anti-inflammatory substances, they are strongly linked to occurrence of CRC [45]. Several miRNAs have been identified as critical regulators of the molecular pathways that connect chronic inflammation to CRC. Upregulated miR-21 is transcriptionally activated by IL-6 via STAT3 and promotes tumorigenesis by targeting the tumor-suppressor PTEN, thereby enhancing PI3K/Akt signaling and sustaining NF-κB activity. This establishes a positive feedback loop that reinforces IL-6 production and STAT3 activation. Similarly, miR-155, induced by NF-κB, promotes Th1/Th17 responses and inhibits apoptosis through suppression of *TP53INP1* and *FADD*. MiR-146a/b modulates Toll-like receptor (TLR) and cytokine signaling by targeting *IRAK1* and *TRAF6*, acting as a negative regulator of excessive NF-κB activation, but becomes dysregulated in the progression from inflammation to dysplasia. Furthermore, upregulated miR-223 directly targets IGF1R and STAT3, modulating insulin-like growth factor and inflammatory pathways, and influences DNA damage response via *ATM* suppression. Collectively, these miRNAs form a regulatory network that integrates inflammatory signaling, immune modulation, and oncogenic transformation, underscoring their functional importance in the inflammation-to-cancer transition in CRC [45,46]. The miR-17-92 cluster, particularly miR-19a and miR-18a, plays crucial roles in regulating inflammation, cell proliferation, and EMT, promoting CRC progression through mechanisms such as the inhibition of tumor suppressors like *TG-2* and *TIA1*, and activation of NF-κB signaling pathways. MiR-19a, specifically, has been shown to induce pro-inflammatory cytokines, which not only contribute to tumor growth but also enhance metastatic potential in CRC. Additionally, miRNAs such as miR-106b, miR-30c, and miR-130a impair autophagy by downregulating genes like *ATG16L1* and *ATG5*, thereby contributing to chronic inflammation and microbial persistence in the gut, further exacerbating CRC risk. In contrast, tumor-suppressor miRNAs, such as miR-124, miR-139-5p, miR-193a-3p, miR-122, miR-192, and miR-495 counteract inflammatory processes by inhibiting NF-κB, STAT3, and Wnt signaling, thereby reducing tumor cell proliferation and enhancing apoptosis. Their downregulation or dysfunction further exacerbates inflammatory responses, increasing the risk of CRC progression, shown in Figure 1 [47,48].

## 4. MicroRNA Dysregulation in Colorectal Cancer

MiRNAs are critically involved in CRC pathogenesis due to their role in regulating gene expression post-transcriptionally [49]. Aberrant miRNA expression drives key tumorigenic processes—uncontrolled proliferation, apoptosis evasion, invasion, angiogenesis, and metastasis—in CRC [50]. Certain miRNAs function as oncogenes (oncomiRs), such as miR-21 and miR-155, which are overexpressed in CRC, inhibit tumor-suppressor genes, and promote tumor growth and metastatic progression [51,52,53]. In contrast, tumor-suppressor miRNAs like miR-143, miR-145, and miR-126 are frequently downregulated in CRC, and their loss fosters cancer progression [54]. Deciphering miRNA dysregulation offers promising diagnostic, prognostic, and therapeutic opportunities in CRC management [50].

### 4.1. Role of MiRNAs in CRC Initiation, Progression, Angiogenesis, Invasion, and Metastasis

MiRNAs play pivotal roles in CRC initiation and progression, with dysregulated miRNAs altering key oncogenic pathways and cellular behaviors [50]. Overexpression of miR-21 is associated with early adenoma–carcinoma transitions and enhances cell proliferation via *PTEN* suppression [55]. Elevated miR-155 promotes CRC cell invasion and metastasis by targeting *APC* and inducing EMT, correlating with advanced stage and lymph node involvement [53]. Tumor-derived miRNAs, such as miR-155, are also implicated in angiogenesis through modulation of claudin-1 and Wnt/β-catenin signaling [50,53]. Tumor-suppressive miRNAs such as miR-34a, miR-200c, and miR-200a have been shown to inhibit EMT by targeting key regulators including ZEB1/ZEB2 and Notch signaling, while dysregulation of miR-200a, in particular, has been associated with poor prognosis and enhanced metastatic potential in CRC [56,57]. Conversely, loss of suppressor miRNAs (e.g., miR-143/145, miR-126) facilitates invasion and metastatic spread [50,53].

### 4.2. MiRNA Interactions with Epigenetics and Signaling in CRC

MiRNAs engage extensively with epigenetic machinery and key signaling pathways in CRC. The miR-29 family directly targets DNA methyltransferases DNMT3A/B, reducing global DNA methylation and reactivating tumor-suppressor genes [58]. Tumor suppressor miR-34a, under p53 control, represses β-catenin/Wnt signaling and induces apoptosis via SIRT1 inhibition [59]. miR-137 is frequently silenced by CpG methylation in CRC, leading to derepression of targets like Cdc42 and histone demethylase KDM4A, thus promoting cell cycle dysregulation [8]. Epigenetically regulated miRNAs such as miR-206, let-7i, and miR-30c-5p play important tumor-suppressive roles in CRC, with miR-206 silenced via promoter hypermethylation and acting through inhibition of Notch3 and Wnt/β-catenin signaling, while let-7i and miR-30c-5p have been recently linked to chromatin remodeling, EMT suppression, and improved patient survival [57,60]. On the oncogenic side, miR-155 and miR-21 are epigenetically upregulated and impair DNA repair (e.g., *MSH2/6*), connecting miRNA dysregulation to genomic instability [61]. Moreover, miRNAs such as miR-101 may directly influence epigenetic remodeling by targeting enzymes like EZH2, a histone methyltransferase, thereby reactivating silenced tumor-suppressor genes and altering chromatin dynamics [56].

### 4.3. OncomiRNAs and Tumor-Suppressor MiRNAs in CRC

OncomiRNAs and tumor-suppressor miRNAs play pivotal roles in CRC pathogenesis. miR-21, an established oncomiRNA, is frequently overexpressed in CRC, promoting tumor growth and invasion through inhibition of *PTEN* and other tumor suppressors [51,62]. Similarly, miR-155 contributes to tumor progression by targeting *APC*, thereby disrupting Wnt signaling and enhancing proliferation and invasion [53]. In contrast, miR-126 functions as a tumor suppressor by modulating angiogenesis and inhibiting PI3K signaling, with its downregulation associated with poor prognosis [50]. MiR-143 also exerts tumor-suppressive effects by targeting KRAS and ERK5, thereby reducing proliferation [63]. Similarly, miR-206 has been identified as a tumor-suppressive miRNA frequently downregulated in CRC, with its reintroduction reducing cell proliferation and migration through inhibition of Notch3 and c-Met signaling [60]. The dysregulation of these miRNAs reflects their dualistic impact on tumor biology and underpins their potential as therapeutic targets and biomarkers in CRC.

### 4.4. The Influence of MiRNAs on Cancer Cell Proliferation, Invasion, Angiogenesis, and Metastasis

MiRNAs play crucial roles in regulating CRC progression by modulating key processes such as proliferation, invasion, angiogenesis, and metastasis. For instance, oncomiRNAs like miR-21 and miR-155 enhance tumor cell proliferation and invasion by targeting tumor-suppressor genes, including *APC*, thereby promoting CRC aggressiveness [53,62]. Additionally, miR-21 is involved in angiogenesis through upregulation of pro-angiogenic factors, facilitating tumor vascularization [64]. Other miRNAs, including miR-210 and miR-424, are hypoxia-inducible and promote angiogenesis via regulation of HIF-1α and VEGF, thus sustaining vascular growth in hypoxic tumor microenvironments [56]. Conversely, tumor-suppressor miRNAs such as miR-126 and miR-143 inhibit CRC cell proliferation and metastatic potential by downregulating oncogenic pathways and extracellular matrix remodeling [63]. The dynamic interplay of these miRNAs orchestrates CRC pathogenesis, influencing tumor growth, local invasion, angiogenic switch, and dissemination to distant organs [50,59]. These findings underscore miRNAs as pivotal regulators and potential therapeutic targets in CRC management. MiR-30a and miR-145 further contribute to anti-invasive effects in CRC by targeting MMP2/9 and SOX9, reducing extracellular matrix degradation and cell motility [56].

### 4.5. The Application of MiRNA Panels in Diagnostics

The application of miRNA panels in CRC diagnostics demonstrates superior sensitivity and specificity compared to conventional biomarkers such as CEA and CA19-9. A combined panel of miR-21, miR-29, miR-92, miR-125, and miR-223 has been shown to enhance diagnostic accuracy by effectively distinguishing CRC patients from healthy controls and those with benign conditions [50,55,62]. A 2025 mini-systematic review further reinforced these findings, identifying 28 validated miRNAs involved in CRC, 14 of which—including miR-200a, let-7i, and miR-30c-5p—were significantly associated with diagnostic accuracy and overall survival based on TCGA data [57]. Individually, miR-21 and miR-92a are consistently upregulated in CRC, correlating with tumor progression, while miR-29 and miR-125 contribute to tumor suppression and are dysregulated in cancerous tissues [51,58]. Moreover, miR-223 plays a critical role in modulating tumor microenvironment and metastasis [50].

MiRNA panels offer several advantages over traditional protein-based markers. They are highly stable in body fluids such as plasma and serum, enabling non-invasive liquid biopsy approaches [65]. This stability supports repeated sampling for real-time monitoring of tumor dynamics and treatment responses. Importantly, specific circulating miRNA signatures, such as low miR-22 and elevated miR-21 levels, have also been linked to chemoresistance in CRC, particularly resistance to 5-fluorouracil, highlighting their predictive utility in therapy selection [56]. Additionally, miRNA expression profiles can reflect tumor heterogeneity and molecular subtypes, improving personalized diagnosis and prognosis [59]. Panels combining multiple miRNAs address the limitations of individual markers by increasing overall diagnostic robustness and reducing false positives or negatives [50,62].

Compared to CEA and CA19-9, which often lack sufficient sensitivity, especially in early-stage CRC detection, miRNA panels achieve higher diagnostic performance, facilitating early diagnosis and improved patient outcomes [66]. Their application extends beyond initial diagnosis to include disease staging, risk stratification, and potentially predicting metastatic potential [50,58]. Thus, miRNA panels represent a promising, non-invasive alternative for CRC screening and monitoring, with ongoing research aimed at validating and standardizing these assays for clinical use. In the table below, we summarized the diagnostic potential of selected miRNAs in CRC. Table 1 summarizes the diagnostic performance of selected miRNAs and panels in CRC.

## 5. MiRNAs as Diagnostic Biomarkers

MiRNAs are increasingly recognized as promising non-invasive biomarkers in cancer diagnostics, including CRC. However, their functional role is context dependent. MiRNAs may act either as oncogenes or tumor-suppressor genes, depending on the tumor type and tissue involved [67].

One of the primary advantages of miRNAs is their detectability in non-invasive biological samples, such as blood, plasma, serum, urine, or stool. Their remarkable stability is particularly notable as miRNAs are resistant to enzymatic degradation, especially when circulating freely or encapsulated in exosomes [12].

### 5.1. MiRNAs with Diagnostic Potential

In blood-derived specimens (including plasma and serum), several miRNAs, such as miR-21, miR-139-3p, miR-135a-5p, and miR-320a, can be effectively detected. Among these, miR-21 stands out for its high sensitivity and specificity in CRC diagnostics (AUC = 0.919). Stool-derived miRNA detection represents a promising alternative to immunochemical fecal occult blood tests (iFOBT), especially given the low detection rates of pre-invasive lesions. Stool samples have been shown to reliably detect miR-29a, miR-223, miR-224, miR-106a, and miR-135b, with miR-106a displaying particular diagnostic potential even in patients with negative iFOBT results [12].

Exosomal miRNAs isolated from serum are also gaining importance. Molecules such as miR-21 and miR-6803-5p have been associated with the regulation of oncogenic processes and may serve as integral components of molecular diagnostic panels for CRC [12].

### 5.2. Application of MiRNAs in Non-Invasive Samples

Recent studies focus primarily on miRNAs with reduced expression in colorectal cancer, which function as tumor suppressors. Some of these miRNAs exhibit well-characterized mechanisms of action directly influencing metabolic pathways, cell cycle control, apoptosis, and mitochondrial biogenesis. For instance, miR-138-5p regulates CRC progression through the MCU/ROS pathway and affects mitochondrial dynamics, whereas miR-4461 modulates vesicular transport by inhibiting COPB2 expression [57]. Similarly, miR-449a exerts its suppressive effects by activating the p53 pathway and also downregulating multiple oncogenes, such as *MYC* and *HDAC1*. Other miRNAs, including miR-452-5p, miR-215-5p, and miR-519 d-3p, although not fully understood mechanistically, have shown clinical relevance. Altered expression levels correlate with disease progression or patient survival, highlighting their diagnostic and prognostic potential [57]. Regarding oncogenes, studies most frequently focus on markers such as miR-21, miR-17, and miR-410-3p [56,68]. Among these, miR-21 is the most extensively studied, exerting its oncogenic effects through the regulation of key targets such as CCND1, PTEN, TGF- βR2, and PDCD4, ultimately leading to increased levels of β-catenin and activation of the Wnt signaling pathway [69].

Numerous studies have investigated plasma levels of miR-21 and miR-145 in clinical cohorts, specifically analyzing samples from 30 CRC patients and 30 healthy controls [70]. MiR-21 levels were more than fourfold higher in CRC patients (*p* < 0.001), especially in cases with proximal colon tumors, indicating its potential as an early diagnostic biomarker. Conversely, a tumor suppressor miR-145 was significantly downregulated, by more than twofold (*p* < 0.001), in CRC patients [70].

MiR-17, another frequently reported oncomiR, promotes tumor cell growth and proliferation by suppressing the tumor-suppressor gene *RND3*. Its active form, miR-17-5p, has been significantly associated with metastatic progression [56].

This represents a paradoxical case, as despite the demonstrated oncogenic activity of miR-17, the study also identified an association between elevated miR-17 expression and a reduced risk of lymph node metastasis in CRC [71]. The authors analyzed miR-17 levels in two sets of biopsy samples: from patients with regional lymph node metastases and from those without metastases. RT-qPCR analysis revealed that higher miR-17 expression was observed in samples from patients without lymph node involvement, suggesting a correlation between increased miR-17 levels and a lower risk of metastatic spread [71].

MiR-410-3p has been shown to play a pivotal role in EMT process. Its function involves the downregulation of *ZCCHC10* and subsequent activation of the NF-κB pathway, thereby enhancing cell migration and invasion in CRC [68]. Additionally, emerging studies highlight the importance of miRNAs with dual functions, such as miR-873. In colorectal cancer, miR-873 targets *ELK1* and *STRN4*, suppressing metastasis and exhibiting tumor-suppressive properties. In contrast, in lung adenocarcinoma, miR-873 has been shown to promote migration and proliferation, indicating its oncogenic role in a tissue-specific context [56]. To identify the most robust and diagnostically relevant miRNAs, an increasing number of studies focus on eliminating those miRNAs whose diagnostic metrics—typically assessed using AUC, sensitivity, or specificity—do not outperform currently employed screening methods. At the same time, research is being expanded to investigate miRNAs that exhibit promising diagnostic potential for future clinical applications. Another study employed a three-phase design comprising discovery, technical validation, and clinical validation. In the discovery phase (30 CRC patients and 32 with advanced adenomas), over 200 miRNAs were identified as significantly upregulated in tumor tissues (FDR < 0.05; FC ≥ 1.5) [72]. From these, 21 miRNAs with the highest overexpression were selected for further validation. In the second phase (n = 78), three stool-detectable miRNAs were prioritized: miR-421 (inhibiting *CASP3* expression; AUC = 0.77), miR-130b-3p (suppressing *CHD9*; AUC = 0.71), and miR-27a-3p (modulating *BTG1*; AUC = 0.69), all genes involved in apoptosis and cell cycle regulation [72,73]. The detection of these miRNAs in the stool of CRC patients suggests active secretion by tumor cells into the intestinal lumen. Notably, the diagnostic performance of a combined panel—miR-421, miR-27a-3p, and fecal hemoglobin—was high (AUC = 0.93), significantly surpassing that of hemoglobin alone (AUC = 0.67) [72]. Yet another study focused on exosome miRNAs isolated from the blood of 88 CRC patients [74]. Using the LASSO algorithm, they identified five miRNAs with high diagnostic potential: hsa-miR-126 and hsa-miR-141 (AUC = 1.000), hsa-miR-139 (AUC = 0.993), hsa-miR-29c (AUC = 0.987), and hsa-miR-423 (AUC = 0.801). These miRNAs showed distinct expression patterns compared to healthy controls, and their links to genes involved in angiogenesis, cell migration, and microsatellite instability underscore their biological significance in CRC progression [74]. To provide a concise overview, Table 2 summarizes selected CRC-related miRNAs, including their molecular targets, biological roles, sample types, and diagnostic AUC values.

### 5.3. Sensitivity and Specificity of Selected MiRNAs in CRC Diagnosis: A Comparative Analysis with Other Diagnostic Methods

**Figure 2:**
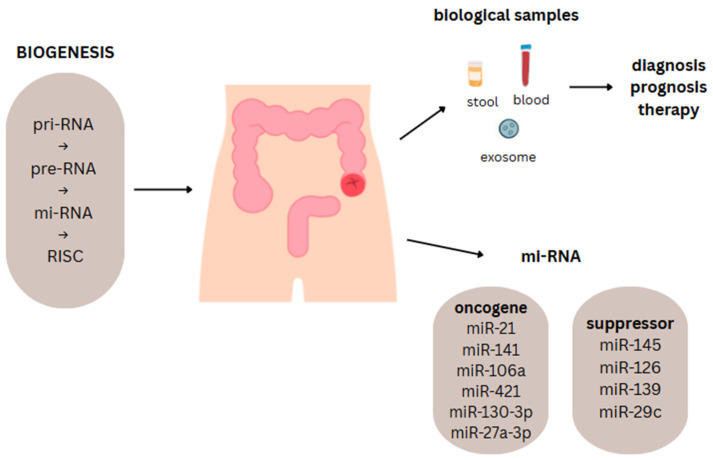
Summary of miRNA types and clinical applications.

The detection of miRNAs in blood serum represents an alternative to conventional screening methods such as colonoscopy, the fecal immunochemical test (FIT), or carcinoembryonic antigen (CEA) measurement. The use of miRNAs can simplify the diagnostic workflow and increase population-wide acceptance of screening protocols, particularly when compared with the more invasive colonoscopy procedure [86,87] (Figure 2).

Colonoscopy, regarded as the gold standard in CRC diagnostics, exhibits very high specificity (97%) and overall diagnostic accuracy of 93% in detecting type V lesions, according to the Kudo Pit Pattern classification. However, its sensitivity in this context is limited to 40%, which reduces its efficacy in identifying a subset of cancer cases [88]. Moreover, for early-stage lesions classified as types III/IV, the detection rate is only 4–5% of all neoplastic changes, further indicating the limited utility of colonoscopy in early disease stages [88].

Regarding the serum marker CEA, commonly used in CRC diagnostics, the sensitivity is 72.27%, and specificity 71.43% [89], reflecting moderate diagnostic value and a considerable risk of both false-positive and false-negative results.

The FIT, especially when implemented using the OC-Sensor system, demonstrates significantly higher specificity (94%) and slightly improved sensitivity (74%) compared to CEA, establishing it as an effective screening tool [90].

However, the most promising outcomes have been observed for circulating miRNAs. A meta-analysis of diagnostic miRNA panels (including miR-15b, miR-21, and miR-31) indicated a sensitivity of approximately 95% and a specificity of 94% [87]. These values exceed those of both the FIT and CEA and are comparable to, if not superior to, colonoscopy in terms of specificity, while offering markedly improved sensitivity.

Various biological materials—stool, plasma, and blood—enable effective detection of miRNA deregulation associated with CRC. Biomarker combinations, such as pairing miRNA panels with fecal hemoglobin measurement, enhance diagnostic accuracy and may form the basis of future, more sensitive testing strategies. These approaches represent a forward-looking alternative to current screening methods, which are not always effective.

## 6. MiRNAs as Prognostic and Predictive Biomarkers

MiRNAs have proven to be a promising diagnostic method to detect CRC in patients, emphasizing the importance of identifying their molecular targets and understanding their impact on metabolic pathways. Nevertheless, there is evidence that they could also play a substantial role in the process of treatment, by inducing chemoresistance in cancer. Anti-cancer therapy resistance poses a serious problem, and specific mechanisms and pathways inducing it should be identified. In this regard, miRNAs might be of significant value, due to their ability to regulate the expression of genes involved in drug response [91].

### 6.1. MiRNA in Response to CRC Treatment

Standard types of chemotherapy for CRC include FOLFOX (5-fluorouracil, leucovorin, oxaliplatin) and FOLFIRI (irinotecan, folinic acid, 5-fluorouracil) [92]. Particular miRNAs were observed to correlate with the treatment response and prognosis for the patient. Dysregulation of miRNAs expression in CRC metastatic patients non-responding to irinotecan-based therapy was found in a cohort study by Pliakou et al. A total of 17 of miRNAs from miR-181 family were found to be upregulated, and 18 of let-7 family were downregulated. Assessing the levels of these two miRNAs families might provide us with a prognosed inadequate response of patients to chemotherapy [93].

A prospective cohort study by Toledano-Fonseca et al. identified circulating miRNAs with predictive value for response to FOLFIRI plus aflibercept in metastatic CRC. Among them, hsa-miR-33b-5p posed the strongest discriminatory potential between patients responding and non-responding to therapy. Its combination with VEGF-A levels was even more accurate in patient stratification. Additionally, higher circulating levels of hsa-miR-33b-5p correlated with significantly better progression-free survival (9 versus 6 months), indicating it as a valuable biomarker in favorable response to this kind of treatment [94].

Exosomal miRNA has also been noticed as a potentially important biomarker, due to its wide presence in body fluids and high stability, along with easy obtainability [95]. A study by Han et al. investigated plasma exosomal miRNA expression profile correlating with oxaliplatin-based chemotherapy resistance in CRC. Results include a panel of exosomal miRNAs, including miR-100, miR-92a, miR-16, miR-30e, miR-144-5p, and let-7i, that potentially differentiates responsive and non-responsive patients to this treatment more accurately than traditional tumor biomarkers (CEA and CA 19-9). This result poses an important future prospective to use miRNA in order to monitor patient response to treatment [96]. In a different study, a panel of serum exosomal miRNAs containing miR-21-5p, miR-1246, miR-1229-5p, and miR-96-5p were found to have the ability to also distinguish the chemotherapy-resistant (oxaliplatin and 5-fluorouracil) group of advanced CRC patients, potentially promoting chemosensivity in this group by targeting these miRNAs [97]. Interestingly, miR-153 upregulation was reported to play a dual role in the progression of CRC: not only does it enhance cellular invasiveness (by inducing matrix metalloprotease enzyme 9 production), but it also plays a role in platinum-based chemotherapy resistance (by inhibiting the Forkhead transcription factor) [98].

High levels of miR-17-5p through regulating the expression of *PTEN* contribute to chemoresistance to CRC chemotherapy. In this regard, chemotherapy might not pose an ideal method of treatment for this patient, indicating that surgery might be a better therapeutic option [99].

All the abovementioned evidence suggests implicating evaluation of miRNAs levels in patients could be of significant value, by estimating response to chemotherapy and offering the most favorable therapeutic option.

### 6.2. Prognostic Value of MiRNA and Its Association with Clinical Outcome

TNM staging classification is still the most commonly used prognostic tool in CRC. However, it does not include the expression of biomarkers in the tumor, such as BRAF, KRAS, and CDX2, which often are strong prognosis indicators [100].

Regarding this area, a study by Rong et al. developed a model of six miRNAs (hsa-miR-1245a, hsa-miR-3682, hsa-miR-4444-2, hsa-miR-5683, hsa-miR-33b, and hsa-miR-152), accurately predicting 5-year survival probabilities for CRC patients, with enhanced predictive ability than TNM stage (AUC: 0.805 vs. 0.694) [101].

Worth mentioning is the fact that specific miRNAs deregulation has been associated with the occurrence of metastasis, such as miR-345 [102], miR-129-2 [103], miR-96-5p [104], and miR-200c [105]. Other factors associated with poor survival and metastasis in CRC are a high expression of miR-185 and low expression of miR-133b, found in the study by Akçakaya et al., evaluating 50 snap-frozen primary sporadic colorectal tumors [106].

MiR-21 was one of the earliest discovered mammalian microRNAs and concluded to play a role in not only CRC diagnosis but also prognosis. High MiR-21 expression was found to be associated with poor therapeutic outcomes, including more rapid disease reoccurrence in patients with TNM stage III colon cancer who received adjuvant chemotherapy and poor overall survival [107]. However, in a different study, no correlation between better survival and high levels of miR-21 of rectal cancer was found [108]. Additionally, miR-21 overexpression was proven to lead to drug resistance to topoisomerase inhibitors (SN-38, doxorubicin, and etoposide/VP-16) in colorectal cancers [109]. A study by You et al. found a positive correlation between high expression of miR-21 and the degree of malignancy of patients, but a negative correlation with survival [110].

A prospective observational cohort study by Mjelle et al. investigating circulating small RNAs as prognostic biomarkers identified miR-320 family as the strongest independent prognostic marker. High baseline levels of these miRNAs were associated with poor survival. Importantly, this study also reported notable post-chemotherapy changes in serum miRNAs and other small RNAs, with distinct expression profiles between responders and non-responders [111]. These outcomes further emphasize the potential of serum miRNA alterations as dynamic biomarkers for treatment response and prognosis.

Table 3 highlights some of the microRNAs whose expression levels are linked to prognosis, metastasis, and treatment response in CRC.

Importantly, miR-7 acts as a tumor suppressor in CRC by directly targeting components of the EGFR signaling pathway. Low miR-7 expression was associated as an independent factor of poor survival. Combining miR-7 with cetuximab enhanced its growth-inhibitory effects, suggesting potential therapeutic relevance [112]. Therefore, another clinical application of miRNA emerges: not only does it contribute to personalizing therapy, but it may also actively participate in therapeutic processes, what would be briefly reviewed below.

### 6.3. The Potential for Personalized Therapy

Knowing that downregulated or upregulated miRNAs expression might play a role in pathogenesis and progression of CRC, targeting it might be a potential focus of cancer treatment. For example, in a study by Zhang et al. [117] overexpression of miR-223 was correlated with CRC, and even more with the high-grade types (Dukes C and D). Subsequently cancer cells were transfected with anti-miR-223, what resulted in decreasing cells’ migration, proliferation, and invasion. Taking into consideration its oncogenic role in CRC through directly targeting p120, miR-223 represents a potential therapeutic target in colorectal cancer [118]. Secondly, a study by Guo et al. [119] investigated the influence of down-regulated miR-137 leading to oxaliplatin resistance in parental cells. The results showed that increased expression of miR-137, through modulating the expression of YBX-1, might sensitize CRC cells to oxaliplatin.

Another treatment personalization involving miRNAs is stratification of high-risk patients. The five-microRNA signature (miR-32, miR-181B, mi-R193B, mi-R195, and mi-R411) was identified as biomarkers to detect lymph node metastases in T1 and T2 colorectal cancer, demonstrating diagnostic accuracy superior to conventional biopsy [120]. This approach might be beneficial in identifying patients who could potentially avoid unnecessary radical colorectal surgery.

These examples highlight the diverse roles of miRNA, that could be incorporated into clinical practice to develop more detailed, patient-customized CRC therapies.

## 7. The Latest Advances and Research Trends

Over the past five years, miRNA research has shifted from exploratory screens to integrated pipelines that link discovery, diagnostics, and intervention. Single-cell small-RNA sequencing measures miRNAs in individual cells and can profile hundreds to thousands of cells per run. Compared with bulk (mixed-cell) assays, it reveals cell-type-specific variants of miRNAs (isomiRs—sequence variants generated by alternative processing or editing) that would otherwise be averaged away, yielding more precise regulatory maps and clearer prioritization of potential drug targets [121]. Rapid, point-of-care testing has advanced in parallel: a 2024 CRISPR/Cas13a lateral-flow assay using MnO_2_ nanozymes enabled amplification-free, visual detection of miR-21 in about ten minutes at sub-picomolar sensitivity, suggesting that true bedside miRNA triage is technically feasible [122]. At the population scale, machine-learning classifiers trained on extracellular-vesicle miRNAs can extract compact signatures with strong discrimination: in the FEVOR project, a gradient-boosting model using fecal EV miRNAs identified a 15-miRNA signature that recognized stage 0-I CRC with an area under the ROC curve (AUC) of 0.94 (where 0.5 indicates no discrimination and 1.0 is perfect), outperforming CEA and CA19-9 measured in the same context [123]. Computational target prediction has also improved; the GraphTar graph neural network, which embeds miRNA-mRNA duplexes with word2vec-style descriptors, reduces false negatives relative to classical seed-match heuristics, thereby easing downstream functional validation [124]. Crucially, functional evidence is increasingly generated in patient-derived systems: in the phase-II PROSPECT-R study, serial NanoString/ddPCR assays guided by patient-derived colorectal cancer organoids implicated circulating miR-652-3p as both biomarker and mechanistic driver of regorafenib resistance, enabling a real-time treatment adjustment [125]. Therapeutic modalities continue to mature: the peptide-conjugated miR-29 mimic MRG-229 reversed collagen programs in rodent and primate models at one-tenth the parent dose and has cleared IND-enabling safety studies [126]. Meanwhile, PEG-liposomal antagomiR-155-5p selectively repolarizes macrophages and reduces joint swelling in murine arthritis without broad immunosuppression, illustrating the safety dividends of nanocarrier delivery [127]. At the same time, a biocompatible microneedle patch loaded with exosomes bearing miR-29b mimics prevented post-infarction cardiac fibrosis in mice, illustrating minimally invasive, tissue-targeted release strategies that avoid systemic exposure [126]. Collectively, advances in single-cell profiling, ten-minute strip tests, AI-guided biomarker discovery, organoid-based validation, and chemically optimized oligonucleotides are coalescing into a practical toolkit that shortens the bench-to-bedside path for miRNA diagnostics and therapeutics.

## 8. Limitations and Challenges

Despite substantial progress in miRNA science, six key barriers continue to slow translation from pre-clinical insight to standard-of-care use. First, biological variability across compartments complicates interpretation: in paired-sample oncomiRs, miR-21-5p, miR-29a-3p, and miR-92a-3p are consistently up-regulated in colorectal cancer tissue but can read ≥ 1 ΔCt lower in matched plasma, which argues against single universal diagnostic threshold [62]. Second, pre-analytical effects—especially haemolysis and processing delays—distort circulating-miRNA profiles. Even mild red-blood-cell contamination or keeping whole blood at 4 °C for ~2 h increases erythrocyte-enriched miRNAs (e.g., miR-451a, miR-16-5p) and shifts dozens of other readouts; routine hemolysis checks and standardized handling before normalization are therefore essential [128]. Third, between-center (inter-laboratory) reproducibility remains limited: multi-site pilots report platform-dependent coefficients of variation near 20% for identical specimens, highlighting the need of shared SOPs, common reference materials, and external quality assessment [129]. Fourth, many diagnostic panels perform well in retrospective case–control cohorts but lose accuracy in prospective validation, demonstrating classic overfitting to the training set. For example, in a 2025 Moroccan case–control study (n = 100), a circulating-miR-21/29a/92a trio reached an AUC of 0.976 but has not yet undergone prospective multi-center validation, leaving generalizability uncertain. To improve generalizability, analysis plans should be pre-registered, model development should use nested cross-validation, and performance should be reported on truly independent test sets with both discrimination and calibration metrics [76]. Fifth, therapeutic safety remains unresolved: the first-in-human phase-I trial of the liposomal miR-34a mimic MRX34 achieved on-target activity, but the trial was halted for immune-mediated toxicities and four deaths, highlighting the need for tighter tissue selectivity (e.g., targeted carriers), proactive immune monitoring, and cautious dose escalation with clear stopping rules [130]. Finally, the growing use of complex algorithms raises practical concerns about bias and opacity. If training data under-represent specific groups (e.g., early-stage disease or particular demographics), risk may be misestimated in those subgroups; moreover, black boxes are hard to audit. We therefore recommend transparent model documentation, reporting subgroup-specific performance, routine bias testing, and external validation prior to deployment [131]. In sum, controlling pre-analytics, standardizing measurement, building and validating models to rigorous, pre-specified plans, and ensuring transparent algorithm reporting are necessary steps before miRNA diagnostics and therapeutics can deliver dependable clinical utility.

## 9. Summary

This review paper provides a comprehensive and up-to-date analysis of the role of miRNAs in CRC, highlighting their emerging utility as diagnostic, prognostic, and predictive biomarkers. CRC remains a leading cause of cancer-related morbidity and mortality worldwide, with early detection and individualized therapy posing major clinical challenges.

MiRNAs, small non-coding RNAs involved in gene regulation, have been identified as critical modulators in CRC development and progression. Their ability to function as either tumor suppressors or oncogenes, and their stable presence in various biological fluids, make them promising candidates for non-invasive biomarkers.

The paper details miRNA biogenesis, mechanisms of action, and interactions with key signaling and epigenetic pathways. Particular attention is given to oncomiRs like miR-21 and miR-155 and tumor suppressors such as miR-143, miR-145, and miR-126, which are dysregulated in CRC and associated with processes like cell proliferation, angiogenesis, invasion, metastasis, and chemoresistance.

Diagnostic applications include the use of miRNA panels detectable in plasma, serum, and stool, with higher accuracy than conventional markers (e.g., CEA, FIT). Prognostically, miRNAs correlate with survival, treatment response, and metastatic potential. Several studies have demonstrated their potential in predicting chemotherapeutic resistance and guiding treatment strategies.

The review also explores the potential of miRNAs in personalized therapy, including targeted miRNA modulation and patient stratification. Recent technological advances, such as AI-driven diagnostics, organoid modeling, and nanocarrier drug delivery, are discussed as enabling tools for clinical translation.

## 10. Conclusions

MiRNAs hold significant promise in revolutionizing colorectal cancer management by enhancing early detection, refining prognostication, and guiding personalized therapies. Their versatility as biomarkers and therapeutic targets stems from their stable detectability in non-invasive samples and pivotal roles in CRC biology.

Although miRNA-based diagnostics are nearing clinical feasibility—with promising sensitivity, specificity, and dynamic monitoring capabilities—several challenges remain. These include biological variability, technical standardization issues, and an incomplete understanding of safety profiles in therapeutic applications.

The integration of advanced sequencing, machine learning, and functional modeling has greatly accelerated progress. However, further multicenter validation, regulatory standardization, and transparency in AI algorithms are essential for clinical adoption.

In conclusion, miRNAs represent a transformative tool in CRC, with the potential to bridge gaps in current diagnostic and treatment strategies. Continued research and clinical translation could position miRNA-based solutions at the forefront of precision oncology in the near future.

## Figures and Tables

**Table 1:** Diagnostic potential of selected miRNAs in CRC.

miRNA	Molecular Mechanism and Clinical Relevance	Diagnostic Utility	Expression Pattern in CRC
miR-21	Promotes tumor growth and invasion by targeting *PTEN*, *KRIT1*, and other tumor suppressors; involved in angiogenesis and metastasis [55,64,65]	High sensitivity and specificity as a circulating biomarker; superior to CEA in early CRC detection [51,55,65,66]	Overexpressed in CRC tissues and plasma; upregulated in tumor and circulating exosomes [51,55,62,64]
miR-92a	Regulates cell proliferation and apoptosis; implicated in Wnt/β-catenin signaling; contributes to tumor progression [54,59]	Effective liquid biopsy marker with high diagnostic accuracy; improves detection when combined with other miRNAs [55,65,66]	Upregulated in CRC tissues and patient plasma; part of miR-17-92 cluster overexpressed in CRC [54,55,64]
miR-29a	Targets genes involved in extracellular matrix remodeling and DNA methylation; influences tumor microenvironment and metastasis [58,59]	Diagnostic potential as part of miRNA panels; useful for distinguishing CRC from benign lesions [58,62,66]	Dysregulated, often downregulated in CRC, but variable depending on subtype and stage [58,59,62]
miR-17-3p	Promotes proliferation and invasion by targeting tumor-suppressor genes and modulating key oncogenic pathways [54,59]	Included in diagnostic panels enhancing sensitivity and specificity in CRC detection [55,66]	Overexpressed in CRC; part of miR-17-92 cluster associated with oncogenic functions [54,59]
miR-223	Modulates immune response and promotes tumor cell proliferation; targets genes involved in cell cycle and apoptosis regulation [49,62]	Potential diagnostic biomarker, particularly in combination with other miRNAs [62,66]	Overexpressed in CRC tissues and plasma; involved in inflammation and tumor progression [49,62]
miR-206	Inhibits tumor proliferation, invasion and migration by targeting Notch3 and Wnt/β-catenin signaling; involved in EMT suppression and apoptosis induction [60]	Potential diagnostic and therapeutic biomarker, especially in combination with methylation status [60]	Frequently downregulated in CRC; low levels correlate with poor differentiation and metastasis [60]
miR-101	Epigenetically silenced in CRC; targets EZH2 and COX-2; modulates histone methylation and inflammatory pathways [56]	Promising biomarker for early CRC detection and therapeutic response prediction [56]	Downregulated in CRC due to CpG island hypermethylation [56]
miR-34a	Induces apoptosis and cell cycle arrest by repressing SIRT1; acts downstream of p53; inhibits Wnt/β-catenin signaling [56]	Recognized diagnostic and prognostic marker; part of p53-related tumor suppressor network [56]	Downregulated in CRC; expression restored by p53 activation [56]
miR-375	Acts as a tumor suppressor by inhibiting proliferation, invasion, and metastasis; targets oncogenic pathways (YAP1, IGF1R) and modulates EMT; low expression linked to advanced TNM stage and poor prognosis [57]	Independent prognostic biomarker; low serum levels correlate with shorter overall survival (OS) and disease-free survival (DFS); potential for non-invasive detection and monitoring [57]	Independent prognostic biomarker; low serum levels correlate with shorter overall survival (OS) and disease-free survival (DFS); potential for non-invasive detection and monitoring [57]

**Table 2:** Selected CRC-related miRNAs, including their molecular targets, biological roles, sample types, and diagnostic AUC values.

Type of MiRNA	AUC	Biological Sample	Molecular Function/Target	Biological Role
miR-21	0.919 [12]	plasma, serum, saliva [12]	PTEN, SPRY2, RECK, PDCD4 [55]	oncogene
miR-135a-5p	0.832 [12]	serum [12]	ERp29 [75]	
miR-29a	0.898 [76]	plasma [76]	KLF4 [50]	
miR-27a-3p	0.690 [77]	stool [77]	Wnt/β-catenin pathway [78]	
miR-141	1.000 [74]	exosomes isolated from blood [74]	PHLPP2 [79]	
miR-423	0.801 [74]	exosomes isolated from blood [74]	possible involvement in p53 pathway, LAMC1 [80]	possibly oncogene
miR-139-3p	0.994 [12]	serum [12]	KRT80 [81]	suppressor
miR-320a	0.886 [12]	serum [12]	Wnt/β-catenin pathway, FOXM1, TWIST1 [82]	
miR-126	1.000 [74]	exosomes isolated from blood [74]	RhoA/ROCK signaling pathway [83]	
miR-139	0.993 [74]	exosomes isolated from blood [74]	Wnt/β-catenin pathway [84]	
miR-29c	0.987 [74]	exosomes isolated from blood [74]	GNA13, PTP4A [85]	

**Table 3:** MicroRNAs whose expression levels are linked to prognosis, metastasis, and treatment response in CRC.

MiRNA	Expression in Tumors	Role in CRC Prognosis	Ref.
miR-7	low	poor survival	[112]
miR-21	high	poor overall survival, poor therapeutic outcome	[107]
miR-21	high	liver metastasis	[102]
miR-96	high	advanced stages of CRC, poor prognosis in patients without distant metastasis at the time of initial diagnosis	[113]
miR-96	low	increased tumor size	[114]
miR-96-5p	low	distant metastasis, independent prognostic factor with respect to cancer-specific survival	[104]
miR-126	low	liver metastasis	[102]
miR-129-2	low	lymph node and liver metastasis	[103]
miR-132	low	progression, poor survival	[115]
miR-133b	low	poor survival and metastasis	[106]
miR-138	low	shorter survival time	[110]
miR-141	high	liver metastasis	[102]
miR-155-5p	high	shortened overall survival and progression-free survival	[116]
miR-185	high	poor survival and metastasis	[106]
miR-200c	high	lymph node metastasis, tumor reoccurrence	[105]
miR-223	high	high grade types of CRC	[117]
miR-345	low	lymph node metastasis, worse histological type	[102]
miR-422a	low	increased tumor size	[114]
miR-584	low	increased tumor size	[114]

## Data Availability

No new data were created or analyzed in this study.

## Abbreviations

The following abbreviations are used in this manuscript:

3′-UTR	3′-untranslated region
AUC	area under the curve
Ago2	Argonaute 2
BTG1	B-cell translocation gene 1
CASP3	caspase 3
CEA	carcinoembryonic antigen
CHD9	chromodomain helicase DNA binding protein 9
CRC	colorectal cancer
DGCR8	DiGeorge syndrome critical region 8
EMT	epithelial–mesenchymal transition
FC	fold change
FDR	false discovery rate
FIT	immunochemical test
GW182	182-kDa protein containing glycine and tryptophan residues
LFAs	lateral flow assays
OncomiRs	oncogenes miRNAs
PABPC	cytoplasmic poly(A) tail-binding protein
PTEN	phosphatase and tensin homolog
Pre-miRNA	primary microRNA
QRT-PCR	quantitative reverse transcription PCR
RISC	RNA-induced silencing complex
ROS	reactive oxygen species
TME	tumor microenvironment
TS-miRs	tumor-suppressor miRNAs
UC	ulcerative colitis
iFOBT	immunochemical fecal occult blood test
mRNA	informational RNA
miRNAs	microRNAs
qPCR	quantitative PCR
